# Up-regulation of syncytin-1 contributes to TNF-α-enhanced fusion between OSCC and HUVECs partly via Wnt/β-catenin-dependent pathway

**DOI:** 10.1038/srep40983

**Published:** 2017-01-23

**Authors:** Ting-Lin Yan, Meng Wang, Zhi Xu, Chun-Ming Huang, Xiao-Cheng Zhou, Er-Hui Jiang, Xiao-Ping Zhao, Yong Song, Kai Song, Zhe Shao, Ke Liu, Zheng-Jun Shang

**Affiliations:** 1 The State Key Laboratory Breeding Base of Basic Science of Stomatology (Hubei-MOST) & Key Laboratory for Oral Biomedicine Ministry of Education, Wuhan University, Wuhan, China; 2 Center of Stomatology, Tongji Hospital, Tongji Medical College, Huazhong University of Science and Technology, Wuhan, China; 3 Department of Stomatology, Liuzhou People’s Hospital, Guangxi, China; 4 Department of Oral and Maxillofacial Surgery, The Affliated Hospital of Qingdao University, Qingdao, China; 5 Department of Oromaxillofacial & Head NeckOncology, School & Hospital of Stomatology, Wuhan University, Wuhan, China

## Abstract

Accumulating evidence implies that cell fusion is one of the driving forces of cancer invasion and metastasis. However, considerably less is still known about the triggering factors and underlying mechanisms associated with cancer-host cell fusion, particularly in inflammatory tumor microenvironment. In this study, we confirmed that inflammatory factor TNF-α could enhance fusion between squamous cell carcinoma cells 9 (SCC-9) and human umbilical vein endothelial cells (HUVEC). Further study revealed that TNF-α could promote up-regulation of syncytin-1 in SCC-9 and its receptor neutral amino acid transporter type 2 (ASCT-2) in HUVEC. Syncytin-1 acted as an important downstream effector in TNF-α-enhanced cancer-endothelial cell fusion. TNF-α treatment also led to the activation of Wnt/β-catenin signal pathway in SCC-9. The activation of Wnt/β-catenin signal pathway was closely associated with the up-regulation of syncytin-1 in SCC-9 and increased fusion between SCC-9 and HUVEC while blocking of Wnt/β-catenin signal pathway resulted in the corresponding down-regulation of syncytin-1 accompanied by sharp decrease of cancer-endothelial cell fusion. Taking together, our results suggest that Wnt/β-catenin signal pathway activation-dependent up-regulation of syncytin-1 contributes to the pro-inflammatory factor TNF-α-enhanced fusion between oral squamous cell carcinoma cells and endothelial cells.

It is well recognized that cell fusion plays a crucial role in a variety of physiological events, such as fertilization, placentation, skeletal muscle formation, and tissue regeneration^1^. Although “cell fusion” theory of tumor metastasis was proposed at the beginning of the 20^th^ century^2^, it had never received extensive attention during the following 100 years. In recent years, there is a growing body of evidence demonstrating the association of cell fusion with cancer progression, including melanoma^3^, breast cancer^4^, colon cancer^5^, *et al*. It is thought that cell fusion provides a profound and unifying explanation for aneuploidy, heterogeneity, metastasis, and drug resistance in cancer^1^,^2^,^5^,^6^,^7^,^8^, and also promotes identification of novel potential targets in cancer therapy. Despite this fact, considerably less is still known about the triggering factors and underlying mechanisms associated with cell fusion in cancer context.

Recently, inflammation has also been suggested as a possible trigger for cell fusion^1^,^9^. Chronic inflammation can led to recruitment of human pluripotent stem cells (HPSCs), mesenchymal stromal cells (MSCs), and cells of the myelomonocytic lineage and subsequently tissue restoration by cell fusion in the late phase of physiological wound healing^10^,^11^. The frequency of cell fusion events was also found about 10 to 100-folds increase in several tissues in chronic inflammatory conditions^11^. Since chronic inflammation is one of the most critical characteristics in tumor microenvironment, it is not difficult to imagine a correlation among inflammation, cell fusion and cancer. Ding^12^ reported that tumor-associated macrophages could promote the progression and metastasis of breast cancer by means of fusion with breast cancer cells. To investigate which factors in the inflamed microenvironment were involved, Mohr^13^ tried various cytokines, growth factors, chemokines and exosomes, and found that the pro-inflammatory cytokine TNF-α, together with hypoxia were strong inducers of cell fusion in human MDA-MB-435 and MDA-MB-231 breast cancer. Our previous study also confirmed that TNF-α could enhance fusion between oral cancer cells and vascular endothelial cells^14^,^15^. However, the signal pathways and effector molecules involved in TNF-α-mediated cell fusion remained poorly understood.

Wnt/β-catenin is one of the most classic signal pathways^16^,^17^. The canonical Wnt pathway regulates the amount of the transcriptional co-activator β-catenin, and thereby controls the crucial developmental gene expression programs^17^. In addition, Wnt/β-catenin pathway has been extensively linked with several pathological processes, including tumor metastasis, epithelial-mesenchymal transition (EMT), stem cell renewal and other diseases^16^,^17^,^18^,^19^. During the fusion of embryonic stem cells with somatic cells, periodic activation of Wnt/β-catenin signal pathway could significantly enhance cell fusion-mediated cell reprogramming^18^. Recently, Matsuura^20^ has reported that β-catenin/BCL9L/TCF4 signal pathway directly targeted the GCM1/syncytin pathway and thereby regulated the fusion of human choriocarcinoma cells. However, the precise molecular activities of Wnt/β-catenin pathway in cell fusion needed deeply clarified.

In the present study, we show that the pro-inflammatory factor TNF-α can activate Wnt/β-catenin signal pathway and thereby enhance the fusion between oral squamous cell carcinoma cells and endothelial cells via up-regulation of fusogenic protein syncytin-1. These results provide new insight into the interaction between oral cancer cells and endothelial cells, demonstrate a signal transduction pathway that links inflammation, Wnt/β-catenin signal pathway and cell fusion in tumor microenvironment, and also predict a novel potential role of chronic inflammation in tumor progression.

## Results

### Spontaneous fusion of SCC-9 with HUVEC cells was enhanced under TNF-α stimulation

**Figure 1:**
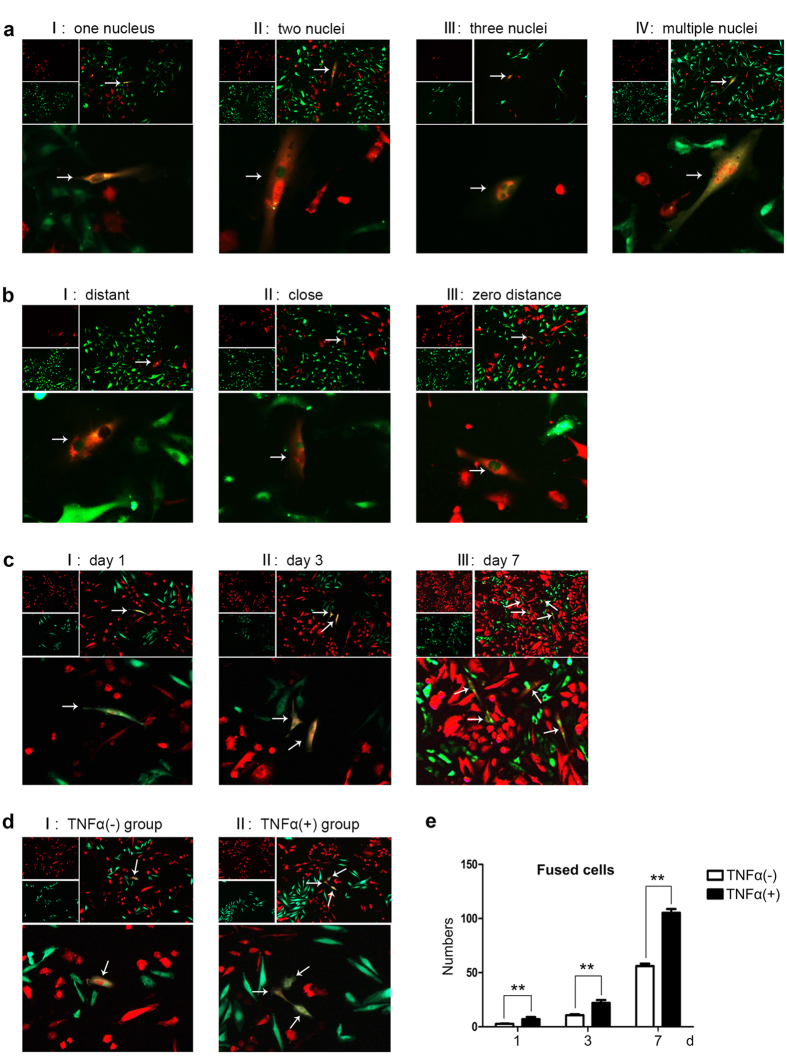
Spontaneous fusion between SCC-9 and HUVEC was enhanced under the stimulation of TNF-α. (**a)** I–IV Representative fluorescence images show the number of nucleus in fusion cells. The number of nucleus is 1, 2, 3 and multiple in sequence from (a I–a IV). (b I–III) Representative images of fluorescence displayed the distance between nuclei in different hybrid cells. (b I) showed that the distance of nuclei in fused cells was relatively far; (b II) showed adjacent; (b III) showed the nuclei fused to one and became zero distance. (**c)** Condition of fused cells after co-cultured for 1, 3 and 7 days. These fused cells appeared healthy. (**d)** Representative images of fluorescence of fused cells between TNF-α group (d II) and normal group (d I). The stimulation of TNF-α increased the percentage of bi-fluorescent cells to 1.78 ± 0.053%, compared with untreated cells (0.748 ± 0.024%). The magnification of images **a**,**b**,**c** and **d** was 100× and 200×, respectively. (**e**) Number of fused cells of TNF-α group and normal group by artificial counting method. The error bars correspond to mean ± SD of at least three independent experiments. *P < 0.05, **P < 0.01, ***P < 0.001.

We co-cultured equal numbers of RFP-labeled SCC-9 and GFP-tagged HUVEC cells for 24 h without any fusogenic reagents. Double fluorescent staining showed that hybrid cells were observed in co-culture system (Fig. 1a–d), demonstrating an existence of spontaneous fusion between human SCC-9 and HUVEC cells. The hybrid cells increased in a culture time-dependent pattern while the rate of spontaneous cell fusion kept constantly (Fig. 1c); Meanwhile, most of hybrid cells appeared healthy and showed no signs of apoptosis or nuclear condensation in co-culture system (Supplement Fig. 1). As shown in Fig. 1a, some of the hybrids contained only one nucleus (Fig. 1aI), while a few contained two, three and/or multiple nuclei (Fig. 1aII–IV, respectively). Different from our previous findings, we noticed in this study that the distance between nuclei was varied in different hybrid cells (Fig. 1bI–III, respectively).

As inflammation microenvironment is strongly linked with tumor progression, we then investigated whether pro-inflammatory factor TNF-α could promote SCC-9 × HUVEC fusion. Under the stimulation of 10 ng/ml TNF-α for 24 h, 10 μg/ml puromycin was used to screen for fluorescence positive cells, and then fused cells were assessed by number counting and FCAS on 1, 3, 7 d, respectively. By counting double fluorescence-positive cells in 3 random fields in a 200 × magnification, we found that cell fusion was enhanced by up to 2~3 (2.18 ± 0.32) folds under the 10 ng/ml TNF-α stimulation compared with untreated cells *in vitro* (Fig. 1c,d). FACS analysis also showed that the stimulation of TNF-α increased the percentage of bi-fluorescent cells to 1.78 ± 0.053%, compared with untreated cells (0.748 ± 0.024%), demonstrating that TNF-α stimulation actively enhance the fusion between SCC-9 and HUVEC cells.

### TNF-α increased expression of fusogenic protein syncytin-1 and its receptor ASCT-2 in SCC-9 and/or HUVEC cells

**Figure 2:**
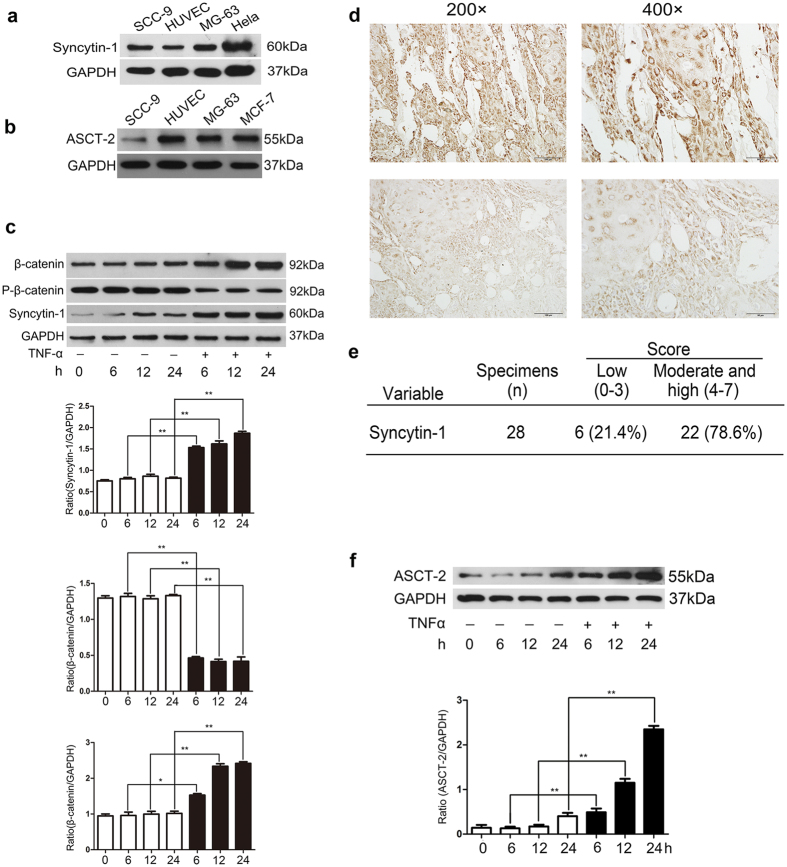
The expression of fusogenic protein syncytin-1 in SCC-9 and its receptor ASCT-2 were increased by TNF-α. (**a**) Protein level of syncytin-1 was evaluated by western blot in SCC-9, MG-63 and Hela cell line. (**b**) Protein level of ASCT-2 was evaluated by western blot in HUVEC, SCC-9, MG-63 and MCF-7 cell line. (**c**,**f**) The difference of syncytin-1 and ASCT-2 expression in 6, 12, and 24 h between TNF-α stimulated group and control group. The expression of syncytin-1 in SCC-9 and ASCT-2 in HUVECs were continuous and dramatic highly expressed compared to the negative control group with 10 ng/ml TNF-α treatment. The expression of phosphorylation of β-catenin (P-β-catenin) decreased sharply in TNF-α group, while the amount of β-catenin increased evidently. (**d**) Positive expression of syncytin-1 in squamous cell carcinoma, 200× and 400×, respectively. The expression of syncytin-1 mainly localized in cellular membrane and/or cytoplasm, and the level of protein expression varied in different samples. (**e**) The level of protein expression was evaluated by score 0–7. 22 out of 28 samples (78.6%) showed moderate and high expression of syncytin-1 and scored 4–7; While others (21.4%) scored 0–3.

Expression of syncytin-1 was detected in surgical specimens from 28 patients with Oral Squamous Cell Carcinoma (OSCC). Immunohistochemistry showed that syncytin-1 was expressed in 26 out of 28 human OSCC specimens (93%) and mainly localized in cellular membrane and/or cytoplasm, but the level of syncytin-1 expression showed different (Fig. 2d); According to our statistics, In these 28 human OSCC specimens, 22 cases of them were detected with a relatively higher expression of syncytin-1 (Scored 4–7), while other 6 cases showed a lower expression (2 of them showed no expression) (Scored 0–3) (Fig. 2e). Western blot using an antiserum against the extracellular domain of syncytin-1 further revealed a 60-kDa band in extracts of SCC-9 as well as HUVEC cells (Fig. 2a), corresponding in size to a syncytin-1 component present in the placenta. ASCT-2 (55 kDa) was also documented in HUVECs and SCC-9 extracts by using western blot analysis (Fig. 2b). Obviously, the expression of syncytin-1 in HUVEC and ASCT-2 in SCC-9 showed evidently lower than syncytin-1 in SCC-9 and ASCT-2 in HUVEC.

To investigate whether TNF-α could regulate the expression of fusogenic protein syncytin-1 and its receptor ASCT-2, we examined the syncytin-1 and ASCT-2 expression in TNF-α stimulation group and control group, respectively. Western blot analysis showed that the constant stimulation of 10 ng/ml TNF-α actually induced a continuous and dramatic increase of syncytin-1 in SCC-9 and ASCT-2 in HUVECs during an observation period of 24 h. Quantitative analysis showed that the ratio of syncytin-1/GAPDH and ASCT-2/GAPDH are significantly higher than the control counterparts at each time-point (Fig. 2c,f), demonstrating that TNF-α stimulation promote the up-regulation of syncytin-1 in SCC-9 and ASCT-2 in HUVECs. It was interesting to note that compared to the control group without any stimulation, the expression of phosphorylation of β-catenin (P-β-catenin) decreased sharply in TNF-α group, while the amount of β-catenin increased evidently.

### Knockdown of syncytin-1 reduced fusion between SCC-9 and HUVEC

**Figure 3:**
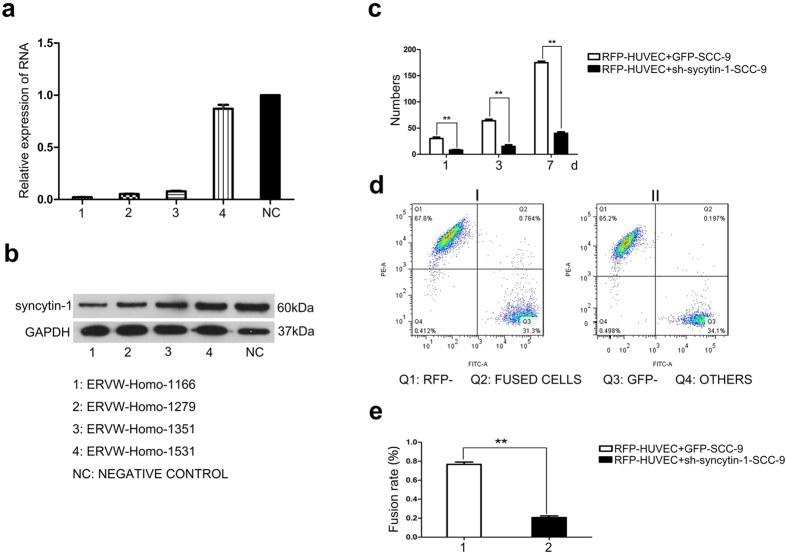
Knockdown of syncytin-1 reduced the fusion between SCC-9 and HUVEC. (**a,b)** The efficiency of four different sh-RNA sequences (sh-RNA1, 2, 3, 4) targeting syncytin-1 and negative sh-RNA sequence which was used as negative control by qRT-PCR and western blot. (**c)** The number of fused cells between SCC-9 and HUVEC when syncytin-1 in SCC-9 was knocked down by sh-RNA by number counting. (**d)** The rate of cell fusion between SCC-9 and HUVEC measured by FACS. When co-cultured SCC-9 whose syncytin-1 was knocked down by sh-RNA with HUVEC, the fusion rate could be 0.196 ± 0.008% compared to the negative control group, 0.768 ± 0.012%. (**e)** The statistical analysis of FACS.

Syncytin-1 has been regarded as an important fusogenic protein in placentogenesis. In this study, we inhibited the endogenous expression of syncytin-1 in SSC-9 cells by short hairpin RNA (sh-RNA) to investigate the biological function of syncytin-1 during cell fusion. Our results showed that knockdown of syncytin-1 significantly inhibited the mRNA and protein expression of syncytin-1 in SCC-9 cells by qRT-PCR assay (Fig. 3a) and western blot (Fig. 3b).

After knockdown of syncytin-1, RFP-tagged HUVECs were co-cultured with GFP-labeled sh-RNA of syncytin-1 in SCC-9 and GFP-labeled SCC-9, respectively. Cell counting analysis showed the transfection of syncytin-1 sh-RNA obviously inhibited the number of fused cells between SCC-9 and HUVEC in co-culture systems on day 1, 3, 7 (Fig. 3c). FACS assay also confirmed an evidently lower fusion rate in the syncytin-1 knockdown co-culture group (0.196 ± 0.008%) than that in control group (0.768 ± 0.012%), showing a significantly statistical difference (Fig. 3d,e). Collectively, our results suggested that interaction of syncytin-1 with ASCT-2 played an important role in mediating the fusion between SCC-9 and HUVEC cells.

### Wnt/β-catenin signal pathway was involved in fusion between SCC-9 and HUVEC

**Figure 4:**
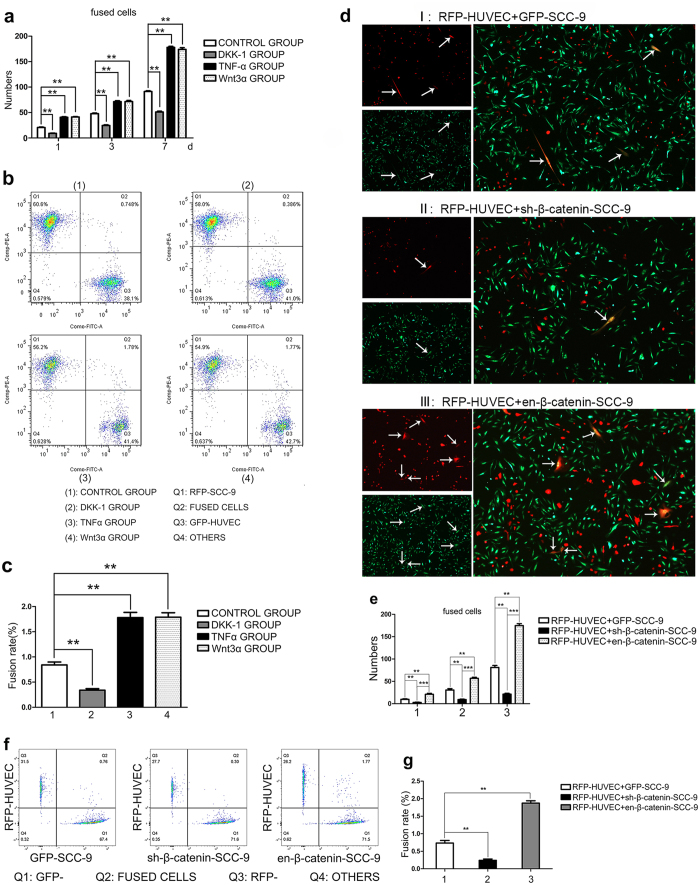
Wnt/β-catenin signal pathway was associated with fusion between SCC-9 and HUVEC. (**a)** The different numbers of fused cells among control group, TNF-α stimulated group, DKK-1 stimulated group and Wnt3α stimulated group by artificial cell counting. (**b)** Different fusion rate among the control group, TNF-α group, DKK-1 group and Wnt3α group by FACS, the fusion rate was 0.748 ± 0.024%, 1.78 ± 0.053%, 0.367 ± 0.016%, 1.76 ± 0.07%, respectively. (**c)** The statistics analysis of fusion rate by FACS of the control group, TNF-α group, DKK-1 group and Wnt3α group. (**d)** The representative fluorescence images of cell fusion between HUVEC and SCC-9 when β-catenin was silenced or/and over-expressed in SCC-9. (d I) showed the representative images of fluorescence when RFP-HUVECs were co-cultured with GFP-SCC-9; (d II) showed the representative images of fluorescence when RFP-HUVECs were co-cultured with sh-β-catenin-SCC-9; (d III) showed the representative images of fluorescence when RFP-HUVECs were co-cultured with en-β-catenin-SCC-9. (**e**) The number of fused cells was evaluated by artificial counting method. The magnification of images d was 100×. (**f**) Different fusion rate between HUVEC and SCC-9 when β-catenin was silenced or/and overexpressed in SCC-9. The fusion rate was 0.30 ± 0.029% when HUVEC was co-cultured with SCC-9 with β-catenin knocked down. When β-catenin was enhanced, the fusion rate between SCC-9 and HUVEC was raised to 1.80 ± 0.033%. (**g**) The statistics analysis of fusion rate by FACS of fusion between HUVEC and SCC-9 whenβ-catenin was silenced or/and overexpressed in SCC-9. The results revealed that Wnt/β-catenin signal pathway was closely associated with fusion between SCC-9 and HUVECs.

Wnt/β-catenin pathway has been reported to play important roles in several molecular events, such as tumor metastasis, EMT, stem cell renewal and cell reprogramming. After activation or inhibition of Wnt/β-catenin signal pathway, fusion between SCC-9 and HUVECs were evaluated by using artificial counting and FACS on co-culture day 1, 3, and 7 in this study. As shown in Fig. 4, the numbers of fused cells increased obviously compared to the control group when the recombinant human Wnt3α, an activator of Wnt/β-catenin signal pathway, was added to the co-culture system. On the contrary, DKK-1, an agonist of Wnt/β-catenin signal pathway, could significantly reduce the fusion events (Fig. 4a). FACS analysis showed that the rate of fusion between SCC-9 and HUVEC reduced to as low as 0.367 ± 0.016% when DKK-1 was added to the co-culture system. However, the fusion rate increased up to 1.76 ± 0.07% when the co-culture system was incubated with Wnt3α (Fig. 4b,c). Both of the DKK-1 group and Wnt3α group showed statistical difference in comparison to the control group.

When SCC-9 cells were pre-treated with β-catenin-specific sh-RNA (sh-β-catenin-SCC-9), the number of double-labeled fluorescent staining cells fell sharply to nearly 1/3~1/2 of the control co-culture group. In accordance with this, the bio-fluorescently-stained cells increased 2~3 folds when β-catenin was overexpressed/enhanced by transfection of en-RNA in SCC-9 (en-β-catenin-SCC-9) (Fig. 4d,e). The FACS results also revealed that the fusion rate reduced to 0.30 ± 0.029% in GFP-SCC-9/RFP-HUVEC co-cultured with presence of sh-β-catenin, but the rate of fused cells would be 1.80 ± 0.033% when en-β-catenin was introduced to co-culture with RFP-HUVEC (Fig. 4f,g). Both of sh- and en- groups showed significant statistical difference in comparison to the control group.

### Activation of Wnt/β-catenin signal pathway controlled the expression of syncytin-1 in SCC-9 cells

**Figure 5:**
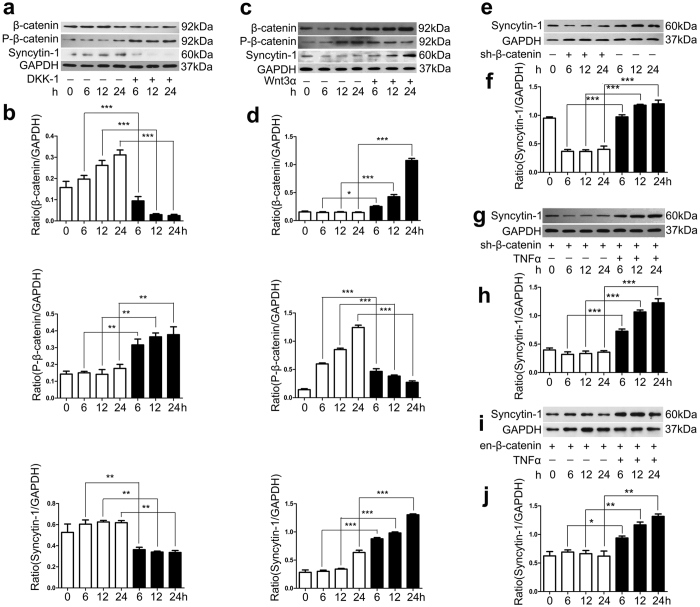
Wnt/β-catenin signal pathway controlled the expression of syncytin-1 in SCC-9 cells. (**a**,**b**) The expression of β-catenin, P-β-catenin and syncytin-1 when SCC-9 was treated with DKK-1, the inhibitor of Wnt/β-catenin pathway, led to the decrease of syncytin-1and β-catenin, but the expression of P-β-catenin was increased. (**c**,**d**) The expression of β-catenin, P-β-catenin and syncytin-1 when SCC-9 was treated with Wnt3α, the activator of Wnt/β-catenin pathway, led to the increased expression of syncytin-1 and β-catenin, while P-β-catenin expression decreased. (**e**,**f**) The expression of syncytin-1 sharply decreased when β-catenin was silenced. (**g**,**h**,**i**,**j**) salvage experiment with 10 ng/ml TNF-α stimulation could restore syncytin-1 expression in either sh-β-catenin or en-β-catenin-treated SCC-9 cells.

To investigate whether syncytin-1 was responsive to Wnt/β-catenin pathway activation, the protein level of syncytin-1 was detected after treatment of the SCC-9 cells with different reagents. As shown in Fig. 5, sustained treatment with DKK-1, an inhibitor of Wnt/β-catenin pathway, led to an obviously decrease of total β-catenin and increase of P-β-catenin which followed by a significant down-regulation of syncytin-1 expression in SCC-9 cells (Fig. 5a,b). After incubation of SCC-9 cells with sh-β-catenin, we also found that syncytin-1 expression presented a time-dependent pattern of decrease and significant difference with control group (Fig. 5e). In contrast, an obvious enhancement of syncytin-1 expression was documented in SCC-9 cells compared to the non-treatment group when the recombinant Wnt3α was used to stabilize and refrain β-catenin from de-phosphorylation and disintegration (Fig. 5c,d). These results suggested that Wnt/β-catenin pathway activation would take part in mediating signal transduction of syncytin-1 in SCC-9 cells. Interestingly, salvage experiment with sustained 10 ng/ml TNF-α stimulation could restore syncytin-1 expression in either sh-β-catenin or en-β-catenin-treated SCC-9 cells, implying that the expression of syncytin-1 is also controlled by some other signal pathway (Fig. 5g,h,i,j).

## Discussion

Cell-cell fusion, an essentially cellular event for normal development and tissue homeostasis, has been involved in cancer progression and attracted extensive attention in molecular oncology in recent years^21^. Our previous study found that oral cancer cells could spontaneously fuse with co-cultured endothelial cells, and the resultant hybrid cells would undergo nuclear reprogramming and acquire a new property of drug resistance and consequently enhanced survival potential^14^,^15^. In this study, we further investigated the mechanisms underlying the enhanced oral cancer/endothelial cell fusion in inflammation microenvironment, particularly focusing on the role of inflammatory factor TNF-α and subsequent Wnt/β-catenin activation-mediated fusogenic syncytin-1 up-regulation during cell fusion.

It is well known that the presence of chronic inflammation is one of the most important characteristics in tumor microenvironment^22^,^23^. More and more studies have demonstrated the association of chronic inflammation and cancer. Recent discoveries of a much broader role of cell fusion in tissue homeostasis and regeneration^21^,^24^,^25^,^26^,^27^, particularly during inflammatory conditions^11^,^28^,^29^, have aroused our great interest to investigate the inflammation as one of the possible driving forces of cancer/host cells fusion. In the present study, we found that the distance between nuclei was varied in different hybrid cells, which may be related to the time point that fusion occurs; We also noticed that the spontaneous fusion between oral SCC-9 and HUVEC was as low as 0.7%~0.8%. However, the frequency of cell fusion between SCC-9 and HUVEC was elevated to as high as 1.5%~1.6% under treatment of sustained 10 ng/ml TNF-α for two weeks. In fact, chronic inflammation has already been reported to result in recruitment of HPSCs, MSCs, and cells of the myelomonocytic lineage and subsequently tissue restoration by cell fusion in the wound healing. The cell fusion rate was also found about 10 to 100-folds increase in several tissues in chronic inflammatory conditions. Together with these findings, our results suggested that TNF-α, a major inflammatory cytokine, might serve as an active trigger factor for cancer-host cell fusion in tumor microenvironment. Importantly, our results revealed a potential link between inflammation, cell fusion and cancer, and provide new insights into the role of inflammation in cancer progression.

Cell fusion is a strictly regulated, poorly understood, multistep process that involves cell-cell recognition, cell-cell adhesion and membrane-membrane fusion^2^,^21^. Syncytin is a captive retroviral envelope protein, and plays an important role in fusions between placental trophoblasts and subsequent formation of syncytiotrophoblasts^30^,^31^. After confirmation of syncytin-1 expression in cancer tissues and SCC-9 cells and its receptor ASCT-2 expression in HUVECs, we investigated the role of syncytin-1 in oral cancer-endothelial cell fusion in this study. When syncytin-1 in SCC-9 cells was down-regulated by sh-RNA technology, assays for cell fusion revealed that knockdown of syncytin-1 significantly inhibited the fusion between SCC-9 and HUVEC in co-culture systems. More importantly, we found for the first time that sustained TNF-α treatment led to a continuous and dramatic increase of syncytin-1 in SCC-9 and ASCT-2 in HUVECs, demonstrating that TNF-α is capable of promoting both syncytin-1 and ASCT-2 expression. Taking together, these results concluded that some inflammatory cytokine, such as TNF-α, might promote cancer-endothelial fusion through up-regulation of fusogenic protein syncytin-1 expression.

Wnt/β-catenin is one of the most classic signaling pathways, and has been extensively linked with in tumor metastasis, EMT, cell reprogramming, and cancer stem cells (CSCs)^16^,^18^,^19^. Accumulating evidence suggested that cancer-host cell fusion would undergo “nuclear reprogramming” process, and the cancer-host cell fusion would be a potential origin of CSCs^12^,^24^,^32^. The cell fusion and cancer stem cell theories might represent possibilities that are generally considered distinct and seem to be considered as “linear” events, each sufficient to ultimately produce metastatic lesions. Therefore, we investigated whether Wnt/β-catenin signal pathway would take part in enhancement of SCC-9/HUVEC fusion under the stimulation of TNF-α. Our results showed that TNF-α treatment dramatically led to the activation of Wnt/β-catenin signal pathway in SCC-9. Assays for cell fusion revealed that either sustained treatment with recombinant Wnt3α or en-β-catenin transfection obviously increased the fusion between SCC-9 and HUVEC. In accordance with this, inhibition of Wnt/β-catenin activation by DKK-1 or β-catenin-specific sh-RNA could significantly reduce the number of double-labeled fluorescent staining cells compared to the control co-culture group. Further study observed that treatment with DKK-1 led to an obviously decrease of total β-catenin and increase of P-β-catenin which followed by a significant down-regulation of syncytin-1 expression in SCC-9 cells. After incubation of SCC-9 cells with sh-β-catenin, we also found that syncytin-1 expression presented a time-dependent pattern of decrease. In contrast, an obvious enhancement of syncytin-1 expression was documented in SCC-9 cells when the recombinant Wnt3α was used to stabilize and refrain β-catenin from de-phosphorylation and disintegration. However, salvage experiment with 10 ng/ml TNF-α stimulation could restore syncytin-1 expression in either sh-β-catenin or en-β-catenin-treated SCC-9 cells, implying that the expression of syncytin-1 is also controlled by some other signal pathways. In fact, periodic activation of Wnt/β-catenin signal pathway could significantly enhance cell fusion-mediated cell reprogramming during the fusion of embryonic stem cells with somatic cells^18^. Recently, Matsuura has reported that β-catenin/BCL9L/TCF4 signal pathway directly targeted the GCM1/syncytin pathway and thereby regulated the fusion of human choriocarcinoma cells^20^. Thus, our results together with theses finding suggested that activation of Wnt/β-catenin signaling pathway could control the expression of syncytin-1, and contribute to the enhancement in cancer-endothelial cell fusion induced by the pro-inflammatory factor TNF-α.

**Figure 6:**
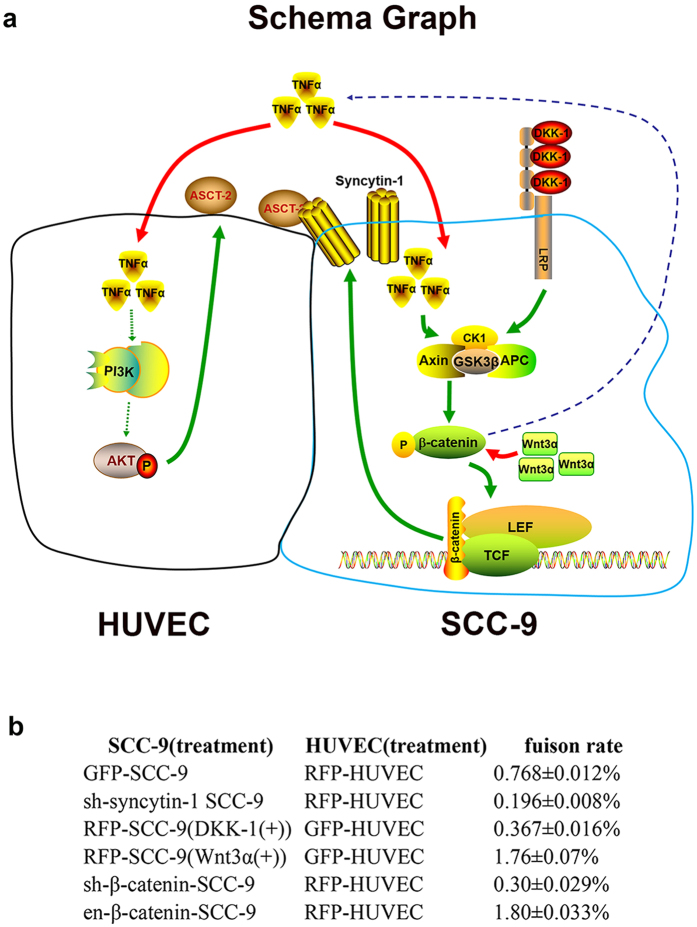
The schema graph and summary of fusion rate between SCC-9 and HUVEC. (**a**) The schema graph of fusion between SCC-9 and HUVEC and the molecular mechanism underlying. When TNF-α was added in SCC-9, the tripolymer of APC, Axin and GSK3β was suppressed, This disrupted phosphorylation/degradation of β-catenin, allowing β-catenin to enrich in the cytoplasm and then enter into nucleus, where it served as a co-activator for TCF/LEF to activate syncytin-1; DKK-1 was the inhibitor of Wnt/β-catenin. DKK-1 could bind to LRP5/6, leading to the activation of the tripolymer (APC, Axin and GSK3β), which on the following would augment the phosphorylation/degradation of β-catenin, and the amount of β-catenin was not sufficient enough to pass into the nucleus, so the expression of syncytin-1 would decreased; Wnt3α could stabilize the status of β-catenin, which would cause the increased expression of β-catenin, afterwards, β-catenin would enter into nucleus and activate TCF/LEF, and then aggrandize the expression of syncytin-1. Our previous studies showed that hypoxia could promote angiogenesis of HUVECs through PI3k/Akt signal pathway. In this study, we preliminary explored and considered that PI3k/Akt signal pathway was closely associated with TNF-α enhanced expression of ASCT-2. There are some reports deemed that some tumor cells could secret TNF-α, we also found that SCC-9 could secret soluble TNF-α, and we thought Wnt/β-catenin could elegantly regulate and control the expression of TNF-α. Further study is needed. When TNF-α was added into HUVEC, it could enlarge the expression of ASCT-2, Next, the ligand, syncytin-1 could bind to the receptor, ASCT-2, and then take part in the control of cell fusion between SCC-9 and HUVEC. ------ steps which had not been confirmed —— steps which had been confirmed. (**b**) The summary of fusion rate with/without stimulus and disposing.

Collectively, the schema graph and the fusion rate of our experiments were showed in Fig. 6. Our present study demonstrates that the Wnt/β-catenin pathway activation-dependent up-regulation of syncytin-1 is involved in the pro-inflammatory factor TNF-α-promoted cell fusion between oral cancer and endothelial cells. These findings not only provide new insights into the links between inflammation and cancer from view of cell fusion, but also uncovere partially the mechanisms underlying the cell fusion in inflammatory tumor microenvironment. The present study may also predict a new therapeutic target for cancer prevention and treatment.

## Methods

### Cell lines and cell culture

SCC-9 cell line was kindly donated by professor Zhuan-Bian, which was purchased from American Type Culture Collection (ATCC, Manassas, VA, US). The SCC-9 was cultured in DMEM/F12 (Hyclone, UT, USA) added with 10% FBS (Gibco, Carlsbad, Calif, USA). Human umbilical vein endothelial cells (HUVECs) were kindly provided by Professor Yi-Fang Zhao and Dr Hai-Xiao Zou; The HUVECs were cultured in EC basal medium 2 (EBM-2, Lonza, Walkersville, MD) which was added with 4% fetal bovine serum(FBS) and EGM-2 growth factor mixture. The HUVECs was restricted in passage 3 to passage 7 when applied in our study. And all of the cells were cultured in an incubator which was kept in 37 °C and contained 5%CO_2_.

### Reverse transcription and qRT-PCR

The total RNA from SCC-9 and HUVECs were extracted via using TRIzol reagent (Takara, Tokyo, Japan). cDNA was developed from total RNA by making use of reverse transcription kit (Takara, Tokyo, Japan). The sequences were: syncytin-1: primer: 5′-GAAGGCCCTTCATAACCAATGA-3′; reverse: 5′-GATATTTGGCTAAGGAGGTGATGTC-3′; The qRT-PCR was performed by the instructions on the SYBR Premix Ex Taq^TM^ II kit (Takara, Japan) and on a 7500 ABI system (Applied Biosystems, USA). All the qRT-PCR data was normalized to GAPDH expression. The tests were repeated at least three times to confirm the consequences.

### Protein extraction and Western blot analysis

Total protein of SCC-9 and HUVECs was extracted by using M-PER (Pierce Inc, USA) supplemented with Protease inhibitor and phosphatase inhibitor on ice. Then the BCA protein assay kit (Thermo Fisher Scientific Inc. USA) was employed to test the quantity of each sample. Afterwards, loading buffer (5×) was added to all of the protein solutions to form mixed liquor. And then solution were heated for 10 minutes at 95 °C. Next, Aliquots of 10 μg of protein were added to 8% sodium dodecyl sulfate-polyacrylamide gel electrophoresis for 30 mins at 60 V and 1 h at 110 V. On the following the proteins were transferred to polyvinylidene difluoride (PVDF) membrane in transfer buffer for 2 h at 200 mA. The membranes were blocked with 5% non-fat milk in Tris-buffered saline which contained 0.05% Tween 20 (the PVDF membrane should be blocked with TBST with 5% BSA when phosphorylated protein was detected) at room temperature for 1 h. Straight after that the membranes were incubated with anti-GAPDH antibody(1:6000) (Proteintech, Wuhan, China), anti-syncytin-1 antibody (1:750, santa cruz), anti-ASCT-2 antibody (1:4000, sigma), anti-P-β-catenin antibody (1:1000, CST), anti-β-catenin antibody(1:20000, epitomics) overnight at 4 °C. Then the bound antibodies were tested by horseradish peroxidase-conjugated, anti-rabbit IgG or anti-mouse IgG (Pierce Chemical, Rockford, IL, USA). Western blot analysis was repeated at least three times to confirm the consequences.

### Immunohistochemistry

28 samples of surgical specimens from patients with oral squamous cell carcinoma were gathered and were fixed with 10% formalin with embedded in paraffin followed. Then antigen retrieval by microwaving was adopted to deparaffinized sections. In the following, the sections were blocked with 10% goat serum. Next, sections were incubated with antibody to human syncytin-1(1:200, santa cruz) and biotin-conjugated secondary antibody and avidin-peroxidase were followed. On the other hand, Nuclei were counterstained with haematoxylin.

For the use of surgical specimens for our research purposes, prior patients’ consents and approval were obtained from the Ethics Committee of School & Hospital of Stomatology, Wuhan University. Written informed consent was obtained from all subjects for publication of this study. All experiments were performed in accordance with approved guidelines of School & Hospital of Stomatology, Wuhan University.

### Block and enhance assay

Wnt/β-catenin signal pathway could be activated by medications such as recombinant human Wnt3α (100 ng/mL) (PeproTech Inc, USA), While to the contrary, DKK-1 (100 ng/mL) (PeproTech Inc, USA) was often used to Block this pathway. So these drugs were elegantly added in SCC-9 to achieve the goal of block or enhance the pathway for 6 h, 12 h and 24 h, respectively.

Recombinant lentivirus was used to silence and enhance/overexpress the expression of β-catenin in Wnt/β-catenin signal pathway. The sequences of recombinant lentivirus which was used to silence the expression of β-catenin: 5′-GCAGGTGTTCTGCTGATATCA-3′. Then 10 ng/mL TNF-α was added to the silenced SCC-9 in 6 h, 12 h and 24 h; On the other side, we divided the normal and silenced cells into two group, and then TNF-α(10 ng/mL) (PeproTech Inc, USA) was sustained for 6 h, 12 h, 24 h. Then we detected the expression of syncytin-1 respectively. Analogously, SCC-9 with enhanced β-catenin was used to test the expression of syncytin-1.

### Cell transduction and co-culture

The lentiviral particles of GFP- and RFP- were purchased from GenePharma, Shanghai, China. The sequence of lentiviral particles of GFP-: 5′-TTCTCCGAACGTGTCACGT-3′; The sequence of lentiviral particles of RFP-: 5′-TTCTCCGAACGTGTCACGTTTC-3′. On the following, the cultured SCC-9 was added with lentiviral supernatants which contained RFP vector. Alike, the cultured HUVEC was added with lentiviral supernatants which contained GFP vector. After transduction for 72 h, the RFP-SCC-9 and GFP-HUVEC were selected with 10 μg/ml puromycin (R&D system, USA). The efficiency of transduction was validated by immunofluorescent microscopy (CarlZeiss, Germany).

### Sh-RNA and en-RNA (Enhanced) assay

The four different sh-RNA sequences (sh-RNA1, 2, 3, 4) targeting syncytin-1, β-catenin and negative sh-RNA sequence (sh-RNA-mock) were purchased from Genepharma (Shanghai, China). The sequence of recombinant lentivirus which was used to silence the expression of syncytin-1: 5′-GCAGCGTCCCCGGAAATATTGA-3′. For constructing human syncytin-1 and β-catenin sh-RNA plasmids, These sequences were inserted into the BamHI/EcoRI sites of pGPU6/GFP/Neo Puro lentivectors. Then 293 T cells were co-transduced. The lentiviral particles were collected and used to infect SCC cells. The infected cells were identifed by 10 μg/ml puromycin. The steps of transfection en-RNA of β-catenin were as same as sh-RNA of β-catenin.

### FACS assay

We co-cultured RFP-SCC-9 and GFP-HUVEC with the mix of 1:1 for 4 groups: group one was cultured with none of the stimulus, while the other three groups were added with TNF-α, Wnt3α and DKK-1. And the results were collected by co-cultured for 1, 3 and 7 days. We also co-cultured sh-syncytin-1 with RFP-HUVEC. The dual-fluorescent positive cells were observed in the fluorescence microscope (Leica, Germany); After co-cultured for 14 days, the dual-fluorescent positive cells were confirmed by FACS (Aria III, BD, USA). We then co-cultured sh-β-catenin and en-β-catenin SCC-9 with RFP-HUVEC for 1, 3, 7 and 14 days, and dual-fluorescent positive cells were counted and confirmed as above.

### Statistical analysis

All statistical analyses were processed by using the Student′s t-test, one-way ANOVA, two-way ANOVA. GraphPad Prism 5.01 (GraphPad Software, USA). FlowJo 7.6.1, FlowJo 10.0 (FlowJo Software, USA). Statistical packages were used to perform statistical data analysis and data were expressed as mean ± standard deviation. The criterion of statistical significance was that *p* values < 0.05. All results were repeated at least three times to confirm the results.

## Additional Information

**How to cite this article**: Yan, T.-L. *et al*. Up-regulation of syncytin-1 contributes to TNF-α-enhanced fusion between OSCC and HUVECs partly via Wnt/β-catenin-dependent pathway. *Sci. Rep.* **7**, 40983; doi: 10.1038/srep40983 (2017).

**Publisher's note:** Springer Nature remains neutral with regard to jurisdictional claims in published maps and institutional affiliations.

## Supplementary Material

Supplement Figure
