# Supplementary material for: Up-regulation of syncytin-1 contributes to TNF-α-enhanced fusion between OSCC and HUVECs partly via Wnt/β-catenin-dependent pathway
Source: Sci Rep. 2017 Jan 23;7:40983. doi: 10.1038/srep40983 (PMC5256027; doi:10.1038/srep40983)
Supplement: Supplement Figure [file srep40983-s1.doc]

**Up-regulation of syncytin-1 contributes to TNF-α-enhanced fusion between OSCC and HUVECs partly via Wnt/β-catenin-dependent pathway**

**Authors:** Ting-Lin Yan ^1^, Meng Wang ^1^, Zhi Xu ^1^, Chun-Ming Huang ^1^, Xiao-Cheng Zhou ^1^, Er-Hui Jiang ^1^, Xiao-Ping Zhao ^3^,Yong Song ^4^, Kai Song ^5^, Zhe Shao ^1,2^ , Ke Liu ^1,2^ , Zheng-Jun Shang ^1,2*^

^1^ The State Key Laboratory Breeding Base of Basic Science of Stomatology (Hubei-MOST) & Key Laboratory for Oral Biomedicine Ministry of Education, Wuhan University, Wuhan, China

^2^ Department of Oromaxillofacial & Head NeckOncology, School & Hospital of Stomatology, Wuhan University, Wuhan, China

^3^ Center of Stomatology, Tongji Hospital, Tongji Medical College, Huazhong University of Science and Technology, Wuhan, China

^4^ Department of Stomatology, Liuzhou People’s Hospital, Guangxi, China

^5^ Department of Oral and Maxillofacial Surgery, The Affliated Hospital of Qingdao University, Qingdao, China.

Ting-Lin Yan, E-mail: yantinglin890929@hotmail.com

**^*^Corresponding author:**

Zheng-Jun Shang, Department of Oromaxillofacial & Head NeckOncology, School & Hospital of Stomatology, Wuhan University, 237 Luoyu Road, Wuhan, 430079, China. Tel: +86 27 87686129, Fax: +86 27 87873260.

E-mail: [shangzhengjun@hotmail.com](mailto:shangzhengjun@hotmail.com)

**Supplement Fig. 1**

**
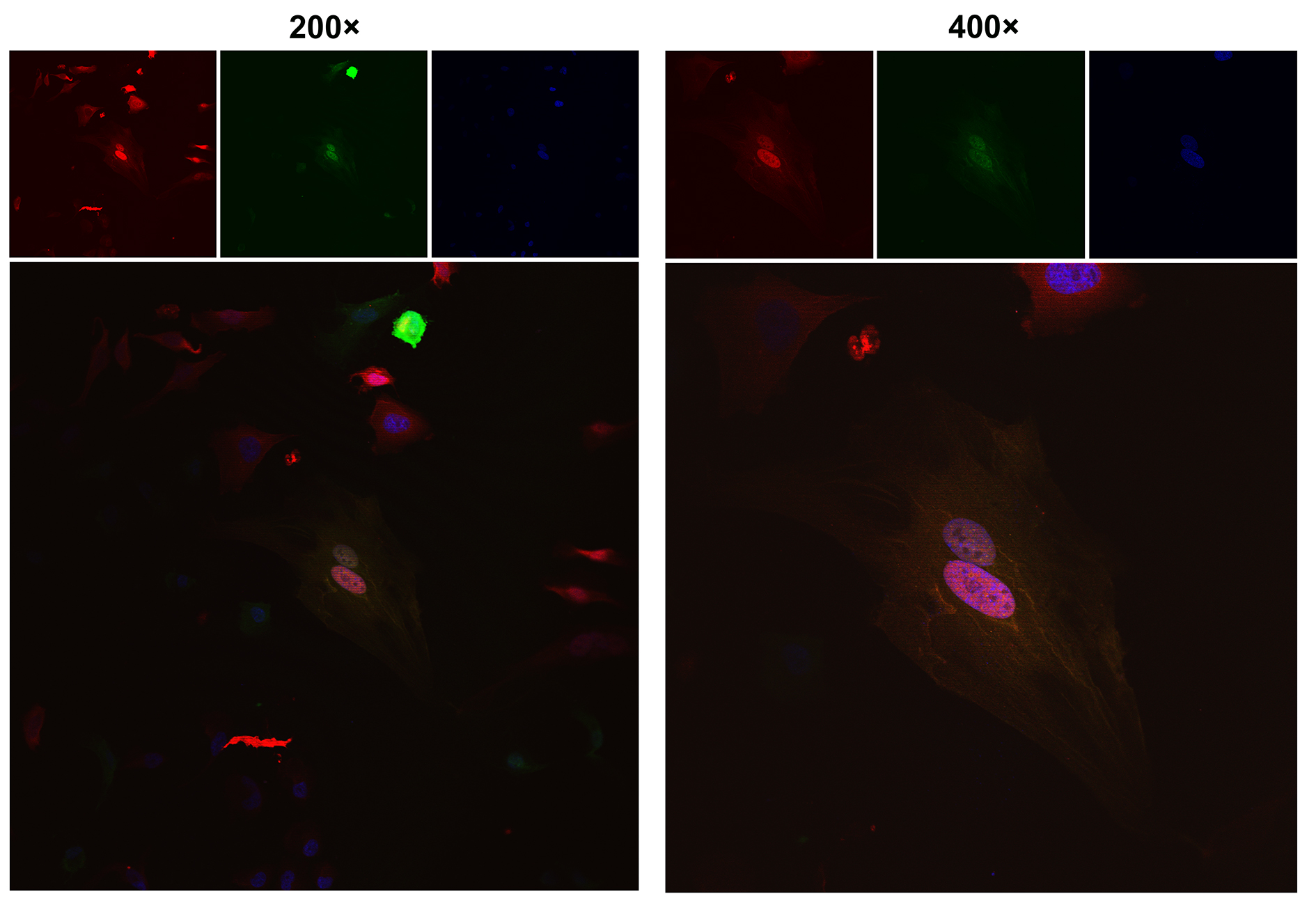
**

**Supplement Fig. 1: Condition of spontaneous fused cells between SCC-9 and HUVEC.**

Representative fluorescence images of fused cells by confocal microscopy. When RFP-SCC-9 was co-cultured with GFP-HUVEC, the fused cells showed orange-like fluorescent. Meanwhile, the cytomembrane of fused cells showed continuous and intact, no cellular shrinkage, foaming or bubbling phenomenon happened. Simultaneously, the nucleolus was apparent, stable stained with DAPI, and the nucleus showed no signs of condensation. The fused cells could be cultured for at least 7-8 weeks.

**Supplement Fig. 2**

**
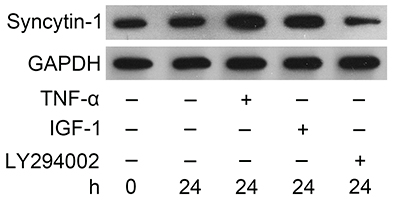
**

**Supplement Fig. 2: The expression of syncytin-1 might partly be controlled by PI3K/Akt signal pathway in SCC-9.**

The expression of syncytin-1 syncytin-1 was detected in 2h with different treatment of reagents. Syncytin-1 expression increased significantly under the sustained stimulation of TNF-α for 24h. When IGF-1, an efficiently stimulus of PI3K/Akt signal pathway was added, the expression of syncytin-1 increased obviously; while on the contrary, the expression of syncytin-1 would decreased evidently when LY294002, the inhibitor of PI3K/Akt signal pathway was persistently used for 24h. From the above, we may conclusion that PI3K/Akt signal pathway may partly control the expression of syncytin-1 in SCC-9.
